# Macrophage-Derived Inflammation Induces a Transcriptome Makeover in Mesenchymal Stromal Cells Enhancing Their Potential for Tissue Repair

**DOI:** 10.3390/ijms22020781

**Published:** 2021-01-14

**Authors:** Inés Maldonado-Lasunción, Nick O’Neill, Oliver Umland, Joost Verhaagen, Martin Oudega

**Affiliations:** 1 The Miami Project to Cure Paralysis, University of Miami Miller School of Medicine, Miami, FL 33136, USA; nvo4@med.miami.edu; 2 Department of Regeneration of Sensorimotor Systems, Netherlands Institute for Neuroscience, Institute of the Royal Netherlands Academy of Arts and Sciences, Amsterdam 1105 BA, The Netherlands; j.verhaagen@nin.knaw.nl; 3 Shirley Ryan AbilityLab, Chicago, IL 60611, USA; 4 Department of Physical Therapy and Human Movements Sciences, Northwestern University, Chicago, IL 60611, USA; 5 Department of Neurological Surgery, University of Miami Miller School of Medicine, Miami, FL 33136, USA; 6 Diabetes Research Institute, University of Miami, Miami, FL 33136, USA; oumland@med.miami.edu; 7 Department of Physiology, Northwestern University, Chicago, IL 60611, USA; 8 Edward Hines Jr. VA Hospital, Hines, IL 60141, USA

**Keywords:** monocytes, immune response, mesenchymal stem cells, growth factors, immunomodulation, regeneration, angiogenesis, survival

## Abstract

Pre-clinical and clinical studies revealed that mesenchymal stromal cell (MSC) transplants elicit tissue repair. Conditioning MSC prior to transplantation may boost their ability to support repair. We investigated macrophage-derived inflammation as a means to condition MSC by comprehensively analyzing their transcriptome and secretome. Conditioning MSC with macrophage-derived inflammation resulted in 3208 differentially expressed genes, which were annotated with significantly enriched GO terms for 1085 biological processes, 85 cellular components, and 79 molecular functions. Inflammation-mediated conditioning increased the secretion of growth factors that are key for tissue repair, including vascular endothelial growth factor, hepatocyte growth factor, nerve growth factor and glial-derived neurotrophic factor. Furthermore, we found that inflammation-mediated conditioning induces transcriptomic changes that challenge the viability and mobility of MSC. Our data support the notion that macrophage-derived inflammation stimulates MSC to augment their paracrine repair-supporting activity. The results suggest that inflammatory pre-conditioning enhances the therapeutic potential of MSC transplants.

## 1. Introduction

Cell transplantation is a therapeutic solution for damaged tissues with little regenerative capability and for inflammatory disorders [1,2,3,4,5,6]. Mesenchymal stromal cell (MSC) transplants support tissue repair in animal models of various conditions by paracrine triggering endogenous repair mechanisms, including the modulation of the local inflammatory response and revascularization [7,8,9,10,11]. Reciprocally, MSCs respond to signals in the damaged tissue environment and choreograph repair events with local cells [12,13,14]. The potential and advantages of MSCs for tissue repair prompted current clinical trials for the treatment of, among others, cardiac damage [4,15], spinal cord injury [2], musculoskeletal repair [16,17] and inflammatory disorders [18,19]. Typically, the effects of MSC transplants on tissue repair and function recovery are limited [20,21,22]. The search for means to augment the effects of MSC transplants on tissue repair is ongoing.

A relatively unexplored method to boost the paracrine activities of MSC transplants is conditioning the cells prior to use. Naturally, circulating signaling molecules specific to a damaged tissue stimulate MSCs to home to the wound and participate in repair [23,24,25,26]. Transplantation strategies typically employ unstimulated, naïve, MSCs implanted directly into the damaged target tissue [1,2]. Conditioning can be applied to MSCs before transplantation (i.e., pre-conditioning or priming) with the aim to ease their introduction and augment their repair interactions in the injury site [27]. Previous evidence showed that MSCs exposed to hypoxia [28], lipopolysaccharide (LPS) [28,29], or cytokines [27,30] alter their secretome in support of repair.

We reasoned that exposure to macrophage-derived inflammation could condition MSCs to increase their repair potential. Inflammation is a fundamental event that prepares damaged tissue for the needed restorative processes [31]. Pro-inflammatory macrophages have a predominant role in early and pathologically chronic stages of inflammation [32,33]. MSCs and macrophages are reciprocally interactive during regenerative wound healing [13,14,34,35]. Here, we investigated macrophage-derived inflammation as a means to condition MSCs through analysis of their transcriptome and secretome and assessed the potential therapeutic value of its use as a beneficial pre-conditioning approach before transplantation.

We found that MSCs conditioned by macrophage-derived inflammation upregulate genes and increase the secretion of growth factors widely associated with promoting blood vessel formation, immunomodulation and tissue repair. We also show that this conditioning method induces the downregulation of pro-survival genes for MSCs and upregulates pro-apoptosis genes. The implications of these later findings and the mechanism of action mediating the overall conditioning effect are under investigation in follow-up studies.

## 2. Results

### 2.1. Study Design

**Figure 1:**
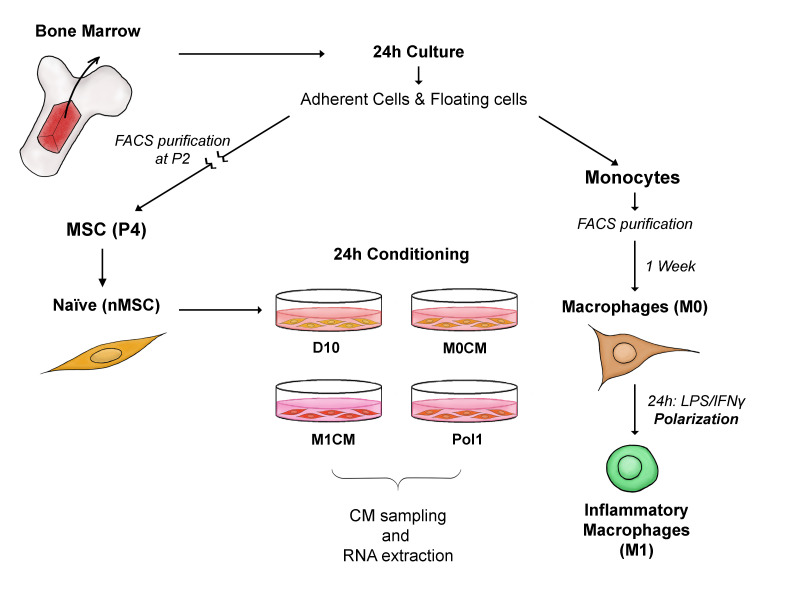
Experimental paradigm. The bone marrow was extracted from femurs and tibias of four rats and cultured on uncoated plastic dishes. After 24 h, mesenchymal stromal cells (MSCs) that adhered to the dish remained in culture and the supernatants were used to sort floating monocytes through FACS. Sorted monocytes were cultured for 1 week on uncoated plastic dishes with R10 medium supplemented with M-CSF to induce differentiation to macrophages. After 1 week, part of the macrophage culture received fresh R10 to remain unpolarized (M0), while the other part received a polarization cocktail with LPS and IFNγ to induce the pro-inflammatory phenotype (M1). In parallel, the MSC culture continued independently. MSCs were purified through FACS at passage 2 (P2) and cultured until P4 on poly(D)-lysine (PDL)-coated dishes. At P4, MSCs were conditioned for 24 h in separate batches with the CM collected from the two macrophage cultures, with the polarization medium used to induce the M1 phenotype and with fresh D10. M0CM, Pol1 and D10 served as controls for the experimental condition M1CM. The CM and RNA was collected from all conditioned MSCs to study the secretome and transcriptome. *Abbreviations*: FACS: fluorescence activated cell sorting; MSC: mesenchymal stromal cell; CM: conditioned medium; (D10, M0CM, M1CM, Pol1) represent the media in which naïve MSCs were cultured for conditioning: D10: DMEM medium with 10% fetal bovine serum and 1:1000 gentamycin; M0CM: M0 macrophage conditioned medium; M1CM: M1 macrophage conditioned medium; Pol1: pro-inflammatory polarization medium.

We investigated the effect of conditioning MSCs with macrophage-derived inflammation by analyzing their transcriptome and secretome. Our experimental design (Figure 1) involved bone marrow-derived MSCs from four rats cultured in parallel providing four biological replicates. Four conditions (i.e., macrophage-derived inflammatory conditioning and three control conditions) were applied to each of these cultures, resulting in sixteen treated MSC cultures (N = 4, k = 4). The control conditions allowed us to evaluate the baseline gene expression of naïve MSCs (D10), the effect of basal macrophage metabolism (M0CM), and the contribution of the macrophage polarization medium (Pol1) on MSC gene expression (Figure 1). RNA samples from each of these treated MSC cultures were prepared for RNAseq. CM samples collected from each of these cultures, and from the differently polarized macrophage cultures, were used in immunoassays. Personnel blinded to the treatments performed all analyses.

### 2.2. RNAseq Analysis

**Figure 2:**
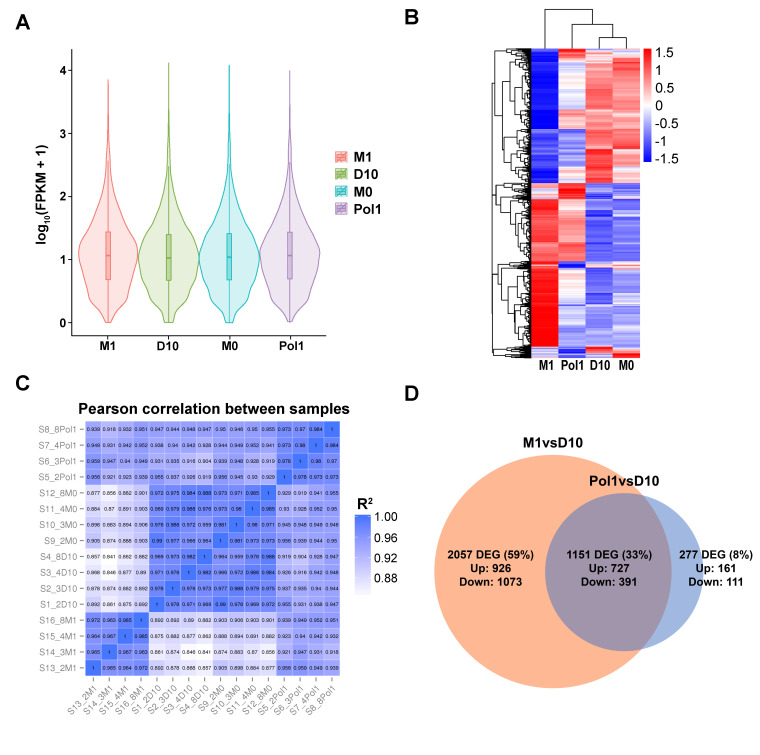
RNAseq overview and quality control. Macrophage-derived inflammatory conditioning of MSCs results in gene expression changes. (**A**) A violin plot shows that the distribution of the level of gene expression is similar across conditions, indicating no abnormalities in the dataset. *x*-axis represents the four conditions, and the *y*-axis represents the mean level of gene expression. Each violin has five statistical magnitudes (max value, upper quartile, median, lower quartile and min value). The violin width shows the gene density. (**B**) Cluster analysis of differentially expressed genes. The log10(FPKM+1) was the unit used for clustering and color scaling. Red denotes genes with high expression levels, and blue denotes genes with low expression levels. The hierarchical tree on the left indicates the relationship between clusters of genes with similar patterns of gene expression. (**C**) Correlation matrix showing the level of similarity in gene expression pattern between samples. The color grading is based on the value of the square of Pearson correlation coefficient. The axes show the sample names (S1 to S16), including the condition they represent. (**D**) Venn diagram comparing the differentially expressed genes (DEG) resulting from the comparisons M1vsD10 and Pol1vsD10. The overlap of both sets represents the genes expressed in both M1 and Pol1 conditions compared to D10. *Abbreviations*: FPKM: fragments per kilobase of transcript sequence per millions base pairs sequenced; D10: MSCs conditioned with DMEM medium with 10% fetal bovine serum; M0: MSCs conditioned with M0CM; M1: MSCs conditioned with M1CM; Pol1: MSCs conditioned with pro-inflammatory polarization medium.

After conditioning, RNA samples were tested for appropriate quantity, quality and purity, confirming their suitability for preparing libraries and performing sequencing (Supplementary Data Section II). RNA sequencing was performed to evaluate gene expression in MSCs after conditioning with M1CM. We obtained an average of 70 million clean reads per RNA sample with 87% mapping to the reference rat genome (Table S1) and assembled into genes. Violin plots showed a uniform distribution of gene expression across cultures, indicating the absence of atypical effects of M1CM conditioning on overall gene expression (Figure 2A).

A total of 26,689 genes were quantified as fragments per kilobase of transcript sequence per million base pairs sequenced (FPKM) and used for the analysis of differentially expressed genes (DEG). We found 3208 DEG under M1CM conditioning, 1428 DEG with Pol1, and 59 with M0CM, relative to those with D10 (Table S2). DEG are visualized in a heat map (Figure 2B) and a co-expression Venn diagram (Figure S1). The full list of DEG is available for reference in the GEO repository under accession GSE161798.

We found a strong correlation (r^2^ > 0.96) between the expression patterns between samples of the same conditioning group and a high similarity between control groups (Figure 2C), indicating high reliability in the experiment. The correlation between D10 and M0CM-conditioned MSCs was 0.96, indicating that basal M0 macrophage metabolism has no relevant effect on MSC gene expression. The correlation between D10 and Pol1 conditioned MSCs was 0.9–0.95, suggesting a difference between samples (Figure 2C). Evaluation of the overlap between the datasets containing the effect of Pol1 and M1 over D10, Pol1vsD10 and M1vsD10, respectively, showed that only 8% of the DEG result from Pol1 conditioning, 59% from M1CM conditioning, and 33% from both M1CM and Pol1 (Figure 2D), suggesting that most of the effect of Pol1 was accounted for within the M1CM vs. D10 dataset. For downstream DE analysis, we will focus on the effects of macrophage-derived inflammation (M1CM), relative to standard D10 medium (D10), on MSC gene expression.

### 2.3. Gene Ontology Analysis

We annotated the DEG between M1 and D10 conditioned MSCs with gene ontology (GO) terms and found 1085 significantly enriched biological processes, 85 cellular components, and 79 molecular functions.

**Figure 3:**
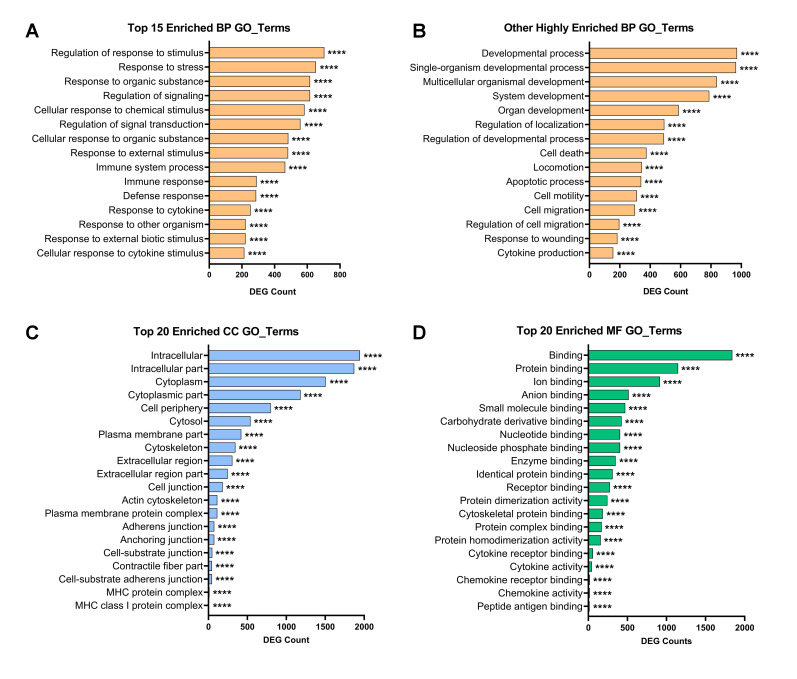
Gene Ontology analysis. Summary of significantly enriched biological annotations with DEG resulting from the comparison M1vsD10. The *x*-axis shows the amount of DEG annotated with each given GO term and the *y*-axis shows the list of GO terms. (**A**) Top 15 of most significantly enriched (lowest *p*(adj)) biological process GO annotations. The common theme is response to inflammation and stress events. (**B**) Other 15 biological process GO annotations with high DEG enrichment that reflect an increased reparative potential of conditioned MSCs, while viability and motility seem to be compromised. (**C**) Top 20 of most significantly enriched cellular component GO annotations, indicating the high level of activity in the cytoplasmic and peripheral regions. (**D**) Top 20 of most significantly enriched molecular function GO annotations, indicating high transcriptomic regulation, intracellular trafficking, and signal transduction. Asterisks represent the statistical value based on the corrected *p*-value resulting from the hypergeometric test (**** indicates *p*(adj) < 0.0005). *Abbreviations*: GO: gene ontology; BP: biological process; CC: cellular component; MF: molecular function; DEG: differentially expressed gene.

**Figure 4:**
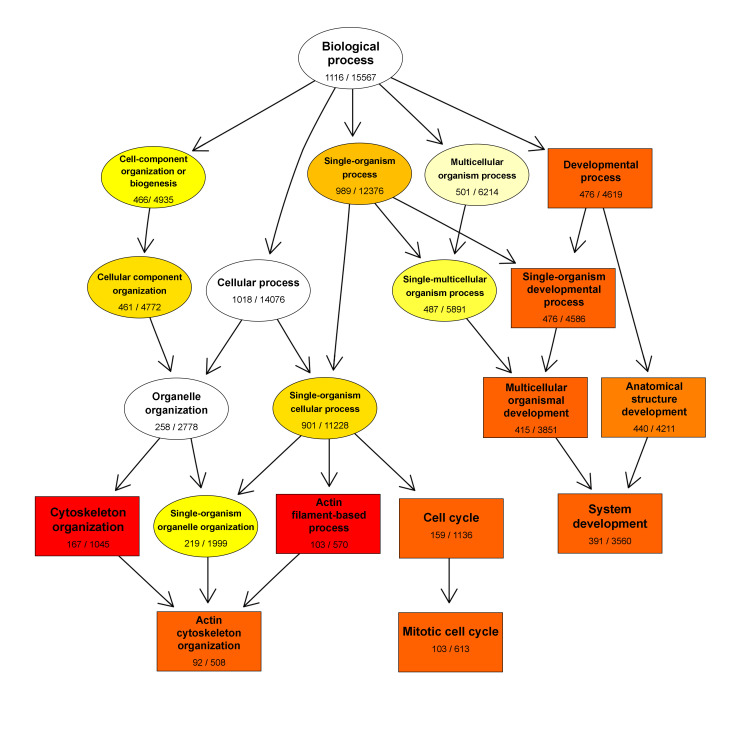
Directed acyclic graph (DAG) of GO terms enriched with downregulated DEG. This DAG represents the hierarchical relationship between biological process GO terms enriched with downregulated DEG from the comparison M1vsD10. Each node represents a GO term and branches represent the containment relationships and the degree of colors represent the extent of enrichment, with black and white ellipses representing non-significant enrichment and yellow to red representing the gradient from higher to lower *p*(adj) values. The numbers under the GO terms are the ratio of downregulated DEG annotated with that term over the number of genes annotated with that term in the reference database. Top 10 of significantly enriched terms are represented in boxes and the rest in ellipses.

Biological processes (BP): The top 20 of the 100 most significantly enriched GO BP terms (Table S3) were related to inflammatory signaling or response to cell-related stimuli (i.e., response to cytokine, immune system process, response to other organism, regulation of signal transduction). The third GO BP term on the list was response to stress, indicating that inflammation triggered stress pathways in MSCs (Figure 3A). Response to stress and defense response are parent terms for 19 other significantly enriched BP terms related to cellular stress, among them oxidative stress and stress activated MAPK cascade. We found upregulated DEG annotated with stress-related terms that are also inflammation mediators or responders: *hif1α, nfkb1, nfkbiz, hgf, mapk, ngf, ido1, irfs*. Besides the genes related to inflammation and defense, we identified four major processes: cell and organism development, response to wounding, cell migration, and cell death (Figure 3B). A directed acyclic graph (DAG) showed that the hierarchy between GO terms enriched with downregulated genes converges towards mitotic cell cycle (Figure 4). Our findings show that inflammatory conditioning activates and enhances MSC-mediated repair programs, while compromising their division through the suppression of actin dynamics and downregulation of survival genes.

Cellular component (CC): Among the 85 most significantly enriched GO CC terms (Table S4), the higher numbers were in Cytoplasm and intracellular, cell periphery, and plasma membrane (Figure 3C), which reflects the high intracellular and extracellular signaling and paracrine activity found in the GO BP terms. Other highly regulated GO CC terms were cytoskeleton, cell–cell junctions, both enriched with downregulated genes (data not shown), and MHC protein complex, enriched with upregulated genes (Figure 3C).

Molecular function (MF): From the 79 most significantly enriched GO MF terms (Table S5), 20 terms related to binding, protein binding, and catalytic activity (Figure 3D). Other GO MF terms related to ion binding, nucleotide binding, integrin binding, and G protein-coupled receptor binding, which is consistent with high transcriptomic regulation, intracellular trafficking, cell migration, and signal transduction, respectively, that occur upon conditioning. Further analysis of the GO MF terms through DAG representation elucidated a statistical relationship between the enriched terms that converged into chemokine activity, which relates to the response to inflammation and cell–cell communication (data not shown).

### 2.4. KEGG Pathway Analysis

**Figure 5:**
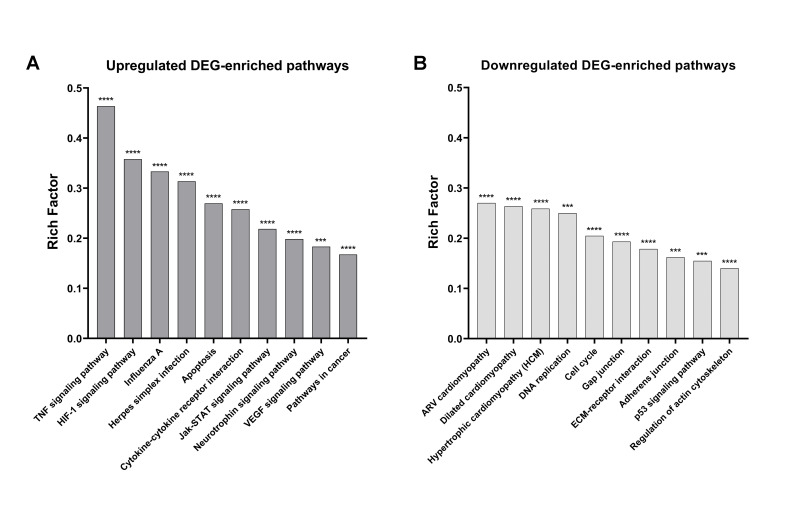
KEGG pathway analysis. Biological pathways enriched with DEGs resulting from the comparison M1 vs. D10. The *x*-axis represents the selected biological pathways from the KEGG database and the *y*-axis represents the rich factor, which is the result of the ratio between the number of DEG annotated with a given pathway in our dataset over the number of genes related to that pathway in the database. (**A**) Top RF biological pathways enriched with upregulated DEG. (**B**) Top RF biological pathways enriched with downregulated DEG. Asterisks represent the statistical value based on the corrected *p*-value resulting from the hypergeometric test (**** indicates *p*(adj) < 0.0005; *** indicates *p*(adj) < 0.005). Abbreviations: RF: rich factor.

The enrichment of biological pathways by our DEG dataset was assessed using KEGG analysis (Figure 5). Because the majority of KEGG pathways present up- and downregulated DEG, we classified them as enriched based on the direction that presented the highest RF. We found 149 pathways significantly enriched with upregulated DEG and 81 pathways significantly enriched with downregulated DEG. The most significant pathways enriched with upregulated DEG (Figure 5A) included Herpes simplex infection (RF = 0.31, padj = 1.12E-24), TNF signaling pathway (RF = 0.46, padj = 2.09E-24), and Influenza A pathway (RF = 0.33, padj = 9.78E-22), which are entries commonly triggered by endogenous inflammation or pathogen infections. The hypoxia inducible factor-1 (HIF-1) signaling pathway was also significantly enriched (RF = 0.36; padj = 9.09E-16).

Other relevant enriched pathways with upregulated DEG included VEGF signaling pathway (RF = 0.18, padj = 0.003), pathways in cancer (RF = 0.17, padj = 1.12E-12), and neurotrophin signaling pathway (RF = 0.2, padj = 1.38E-6) (Figure 5A). These pathways, combined with the significantly upregulated growth factor genes VEGF (*vegf*), HGF (*hgf*), NGF (*ngf*) and GDNF (*gdnf*), among others, indicate an MSC transcriptome in support of repair. In contrast, enriched pathways with upregulated DEG were also apoptosis pathway (RF = 0.27, padj = 1.65E-12) and *jak-stat* signaling pathway (RF = 0.22, padj = 3.41E-9) and enriched with downregulated DEG was the cell cycle pathway (RF = 0.2, padj = 3.13E-7). These last pathways, combined with the downregulated survival gene, B-cell lymphoma 2 (*Bcl2*), suggest a compromised MSC viability.

Pathways enriched with downregulated DEG are involved in MSC mobility included dilated cardiomyopathy (RF = 0.26, padj = 4.23E-8), focal adhesion (RF = 0.17, padj = 4.23E-8), hypertrophic cardiomyopathy (RF = 0.26, padj = 1.81E-7), and arrhythmogenic right ventricular cardiomyopathy (RF = 0.27, padj = 3.13E-7) (Figure 5B). All were characterized by the downregulation of genes involved in actin polymerization and cell junctions. Lists of the top 60 pathways with upregulated DEG (Table S6) and downregulated DEG (Table S7) are provided in the Supplementary Data, and they include links to visualize the corresponding full molecular pathways (e.g., Figure S3). Our KEGG analysis demonstrated that MSCs conditioned with macrophage-derived inflammation undergo metabolic changes that support cell/tissue repair but challenge their survival and mobility.

### 2.5. Secretome

**Figure 6:**
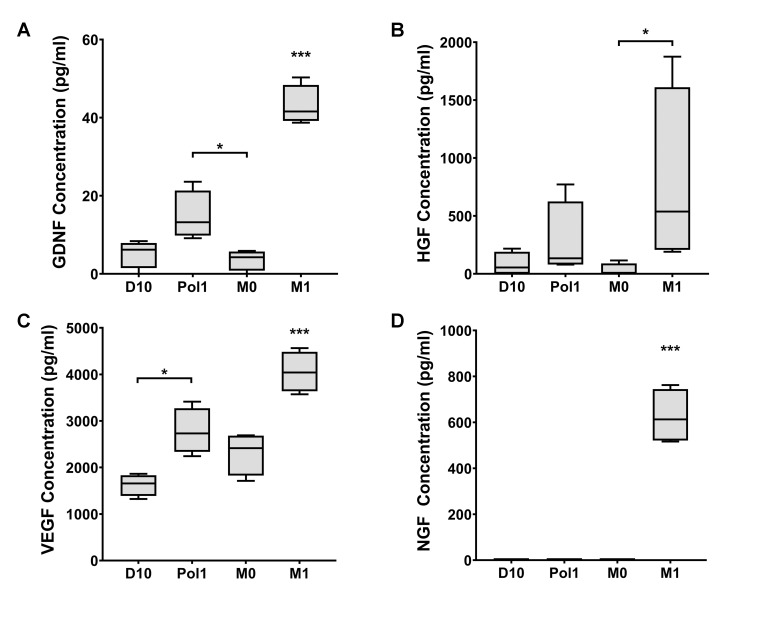
Secretome analysis. Growth factor concentration in MSC conditioned medium after 24 h conditioning was measured with immunoassays. (**A**) Box plot of the concentration of GDNF in MSC CM across conditions. M1CM conditioning induces significantly higher secretion of GDNF compared to the three controls. Pol1 conditioning induces significantly higher secretion of GDNF than M0CM. (**B**) Box plot of the concentration of HGF in MSC CM across conditions. M1CM conditioning induces significantly higher secretion of HGF compared to M0CM. (**C**) Box plot of the concentration of VEGF in MSC CM across conditions. M1CM conditioning induces significantly higher secretion of VEGF compared to the three controls. Pol1 conditioning induces significantly higher secretion of VEGF than regular D10. (**D**) Box plot of the concentration of NGF in MSC CM across conditions. M1CM conditioning induces significantly higher secretion of NGF compared to controls. Asterisks represent the level of significance on one-way ANOVA test (**A**,**C**,**D**) or Kruskall–Wallis test (**B**), with * = *p* < 0.05 and *** = *p* < 0.0005. Error bars indicate minimum and maximum values. Abbreviations: GDNF: glial-derived neurotrophic factor; HGF: hepatocyte growth factor; VEGF: vascular endothelial growth factor; NGF: neural growth factor; CM: conditioned medium; D10: MSCs conditioned with DMEM medium with 10% fetal bovine serum; M0: MSCs conditioned with M0CM; M1: MSCs conditioned with M1CM; Pol1: MSCs conditioned with pro-inflammatory polarization medium.

Using ELISAs, we measured the levels of NGF, GDNF, VEGF-A, and HGF secreted by M1CM-conditioned MSCs compared with controls. Macrophages secrete certain growth factors at different stages of polarization, so we included samples of M0CM and M1CM in the ELISA readings and used the outcome to correct for the background presence of growth factors in the conditioning media. We found that M1CM-conditioned MSCs secreted significantly increased levels of NGF (F (3, 12) = 108.22, *p* = 5.92E-9) (Figure 6A), GDNF (F (3, 12) = 62.65, *p* = 1.33E-7) (Figure 6B) and VEGF-A (F (3, 12) = 23.86, *p* = 2.4 × 10^−5^) (Figure 6C), compared to the controls. Pol1-conditioned MSCs secreted significantly increased levels of GDNF (Means Differenc = 11.2, *p* = 0.03) compared to M0CM-conditioned MSCs, and increased levels of VEGF compared to MSCs in D10 (Means Difference = 1154.82, *p* = 0.013). The level of secreted HGF by M1CM-conditioned MSCs was significantly higher (z = 2.69, *p* = 0.042) compared with M0CM-conditioned MSCs, but not compared with D10 conditioned MSCs (z = 2.24, *p* = 0.149, effect size = 0.79) (Figure 6D). Because the HGF data were not normally distributed, the Kruskal–Wallis test was used (H (3) = 8.463, *p* = 0.037), followed by Bonferroni correction. Together, our data show that macrophage-derived inflammation conditioning of MSCs enhances the secretion of several key repair-supporting factors.

## 3. Discussion

Preclinical studies and ongoing clinical trials have revealed the therapeutic potential of MSC transplants for repair of a variety of tissues. Here, we investigated whether conditioning MSCs with macrophage-derived inflammation would boost their ability to support tissue repair. We showed that macrophage-derived inflammation triggers MSCs to upregulate genes associated with wound healing and enhances the secretion of molecules fundamental to reparative events. We also showed that inflammation-induced changes in the MSC transcriptome challenge their viability and mobility. Further investigation is ongoing to identify the optimal balance between the beneficial and potentially detrimental effects of inflammation on the transplant. Our data support the notion that macrophage-derived inflammation can be employed to condition MSCs prior to transplantation to augment their paracrine repair effects, which may enhance the therapeutic efficacy of MSC transplants.

Conditioning MSCs with macrophage-derived inflammation resulted in increased expression of genes related to, among others, response to inflammation, negative regulation of cytokine release, defense response and response to stress (e.g., *ido1, nos2, il13r, ptgs2, hif1α, nfkbiz*), suggesting that the conditioning potentiated the immunomodulatory ability of MSCs. Cellular stress is considered a molecular adaptation to either restore homeostasis or induce cell death in response to external or internal threats [36,37]. Stress related genes are constitutive genes that maintain cellular homeostasis in normal conditions but are upregulated at the trigger of events, such as inflammation, toxins, temperature, or hypoxia, among others [36,38,39]. In normoxic inflammatory conditions, such as in our cultures, the enrichment of the HIF1α signaling pathway with upregulated DEG, suggests an adaptation to physiological stress, which can be protective for the cell and the surrounding tissue. Our culture conditions include the presence of inflammatory cytokines and LPS, a bacterial endotoxin, which are interpreted by MSCs as alerts of system disruption and trigger the upregulation of *hif1α* HIF1 and *nfkb* NFKB, both master transcription factors associated with immunity, stress, and repair pathways [40,41,42]. Evidence showed that exposure to stimuli triggering cellular stress activates the repair and immune regulatory response of MSCs [10,14,43], resulting in stronger paracrine modulation of the phenotype of nearby macrophages [44,45,46]. The regulation of the macrophage phenotype is important because it coordinates efficient wound healing [47,48,49] and contributes to recovery after damage in various tissues, including cardiac [27,50,51], nervous [52,53], cutaneous [54] and muscular tissues [55]. Local immunomodulation can modify the molecular environment and other immune cells that also have a direct or indirect effect on macrophage phenotype, such as regulatory T cells [56,57]. Proper coordination of immune and stromal cell interactions can also lead to enhanced angiogenesis [58,59]. Increasing the immunomodulatory capacity of MSCs prior to transplantation may render a more efficient transplant, especially for repair of tissues hallmarked by chronic inflammation.

Inflammatory conditioning of MSCs caused an increase in the expression of genes involved in angiogenesis (i.e., *vegf, ang-1, hgf, egf*) and in the secretion of pro-angiogenic factors, VEGF and HGF compared to unconditioned MSCs. Additionally, the VEGF signaling pathway and HIF-1 signaling pathway were enriched with upregulated genes. These results suggested that conditioning with macrophage-derived inflammation augmented the angiogenic ability of MSCs. VEGF is a master growth factor that initiates angiogenesis by inducing vascular endothelial cell proliferation and endothelial tip cell differentiation, which are needed for vascular tube formation [60,61,62]. Previous research showed that treatment with recombinant VEGF promotes revascularization, which correlated with reduced injury expansion in the damaged spinal cord [63] and increased bone regeneration [64]. Angiopoietins are key in increasing maturation and decreasing permeability in newly formed blood vessels [65,66]. HGF, originally a hepatocyte mitogen, is associated with enhanced angiogenesis and blood vessel protection [67]. Aoki and colleagues demonstrated that HGF supports angiogenesis, thereby contributing to the repair and recovery of nervous and cardiac tissues [68,69]. Enhancing the angiogenic effects of MSCs prior to transplantation into damaged tissues may provide better transplant-mediated revascularization, which is important for optimizing tissue repair.

The macrophage-derived inflammatory conditioning of MSC-enriched repair-related annotations, such as tissue development, response to wounding, and neurotrophin signaling, indicated stronger activation of general repair-supporting mechanisms in the conditioned MSCs compared with unconditioned MSCs. Neurotrophins, such as NGF and GDNF, as well as insulin-like growth factors (IGF family), fibroblast growth factors (FGF), and HGF trigger cell survival and axon outgrowth pathways and induce healing in numerous types of tissues [70,71,72,73,74,75,76,77]. Improving local cell survival is typically associated with better repair and recovery [9,68,76]. Interestingly, the term pathways in cancer was enriched with upregulated genes following the inflammatory conditioning of MSCs. Considering the overlap in molecular mechanisms between cancer and regenerative biology (e.g., mTOR pathway, Wnt signaling pathway) [78,79,80,81,82,83], it is possible that genes involved in these overlapping pathways and upregulated in our dataset, such as *vegf, hif-α, hgf, fgf, bmp, wnt,* and *frizzled*, also contribute to repair. Together, our data indicate that the macrophage inflammation-derived conditioning of MSCs trigger stronger paracrine survival mechanisms compared with unconditioned MSCs. Increasing the ability of MSCs to support cell survival prior to transplantation may result in improved transplant-mediated tissue repair.

Our findings revealed that macrophage inflammation conditions MSCs in support of immunomodulation, angiogenesis, and cell survival. These three aspects in wound healing are tightly and reciprocally coordinated, combining into a necessary repair triad for successful outcomes [48,84]. Different phenotypes of macrophages are needed during different phases of angiogenesis [85,86,87,88,89] and have different influences on local cell survival. Angiogenesis is needed for limiting the often-progressive loss of cells and tissue in an injury site. Reciprocally, cell protection supports blood vessel maintenance, and proper vascularization influences macrophage phenotype. Thus, the crosstalk between MSCs and macrophages is essential for successful repair [12,13,14] and the increased immunomodulatory, angiogenic and protective capacity of MSCs following inflammatory conditioning may accelerate the overall therapeutic effects of MSC transplants.

The macrophage-derived inflammatory conditioning of MSCs also caused a decrease in the expression of genes involved in cytoskeleton activity, actin dynamics and cell adherence capacity, which could challenge MSC viability and motility. The possible decrease in viability is in agreement with the finding that exposure of MSCs to the inflammatory molecules, IFNγ and TNFα induces apoptosis [22,90]. The overall impact of the possible attenuation in MSC viability and motility after conditioning with macrophage-derived inflammation is unknown. In general, the viability of transplanted cells in an injury is low, due to different factors, including immune incompatibility causing rejection, lack of substrate leading to anoikis [91,92], or cytotoxic molecules causing apoptosis and necrosis. Promoting transplanted cell survival may lead to improved repair and recovery [35,93,94]. Thus, the attenuation of viability due to inflammatory conditioning may limit the duration of the repair effects of transplanted MSCs. On the other hand, Dazzi and colleagues reported that macrophages in an injury phagocytose apoptotic MSC leads to PGE2 and IDO1 production and more effective immunomodulation than the phagocytosis of live, healthy MSCs [95,96]. This has led to the notion that sacrificial death by MSCs could potentially enhance their contribution to immunomodulation. Another possible advantage of attenuated MSC viability is that spontaneous tumor development by the transplanted cells could be prevented.

The consequences for the attenuated adhesion and motility of MSCs following conditioning with macrophage-derived inflammation are unknown. It is possible that, with direct injection into an injury, a reduced migratory ability will be beneficial because the MSCs will remain where they are needed. On the other hand, with the systemic administration of MSCs, a decrease in cell adhesion and motility could possibly compromise their ability to reach the intended target site [24,25,26,34]. Further research is necessary to more comprehensively understand the in vivo implications of macrophage-derived inflammatory conditioning, and other types of conditioning, of MSCs. Current follow-up studies within our lab are targeted to answering these questions and unravel the molecular mechanisms by which macrophages modify MSC behavior. A better understanding of the molecular relationship between stress, inflammation and repair is necessary to potentially design a more effectively targeted conditioning approach. It is possible that different conditioning methods will need to be used depending on the route of administration and the therapeutic application.

**Figure 7:**
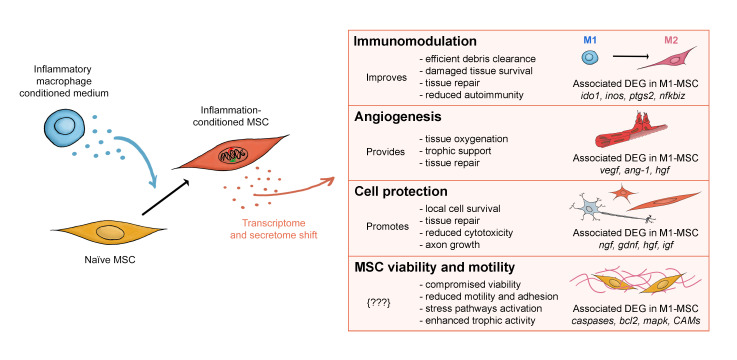
Graphic summary. Macrophage-derived inflammatory conditioning induces changes in the transcriptome and secretome of bone marrow-derived MSCs. Conditioned MSCs have increased expression levels of genes that are known to participate in immunosuppressive and immunomodulatory events (e.g., *ido1*, *nos2, il13r, ptgs2, hif1α, nfkbiz*), suggesting that macrophage-derived inflammation potentiates the immunomodulatory ability of MSCs. Similarly, conditioning induced higher levels of expressed genes (e.g., *vegf*, *ang-1*, *hgf, egf, hif1)* and secreted growth factors known to promote angiogenesis, indicating the potentiation of MSC angiogenic ability. Inflammatory conditioning also increased the expression of genes associated with the promotion of tissue repair and cell survival (e.g., *ngf, gdnf, igf1, bmp).* Given that immunomodulation, angiogenesis and the promotion of cell survival are synergistic events that are essential to tissue repair, our work suggests that macrophage-derived inflammatory conditioning of MSCs before transplantation may enhance the therapeutic potential of MSCs transplants. On the other hand, our data also show that conditioned MSCs show signs of apoptosis, arrested cell cycle and reduced motility, because genes related to cell death and apoptosis are upregulated, while survival genes (i.e., *bcl-2*) are downregulated. The impact of these challenges on the therapeutic potential of MSCs transplants is yet unknown, so further research is needed to better understand and optimize the conditioning method. In the chart, we summarize the sub-phenomena that result from enhancing the respective physiological processes in damaged tissue. The curved arrows, on the left, represent the effect of macrophages’ (blue) or conditioned-MSC’ secretomes on other cells, while the straight black arrow represents a phenotype transition from naïve MSC to conditioned MSC. Abbreviations: MSC = mesenchymal stromal cell; M1 = pro-inflammatory macrophage; M2 = anti-inflammatory macrophage; DEG = differentially expressed genes; M1-MSC = macrophage-derived inflammation-conditioned MSC; {???} = unknown effect on the therapeutic efficacy of pre-conditioned MSC transplants; CAMs = cell adhesion molecule-related genes.

In conclusion, we showed that conditioning MSCs with macrophage-derived inflammation increases the expression of genes involved in immunomodulation, revascularization, and cell survival, which are vital for tissue repair (Figure 7). These encouraging results revealed that the inflammatory conditioning of MSCs might boost the therapeutic strength of MSC transplants for a variety of damaged tissues, which has widespread clinical relevance. A benefit of using macrophage-derived inflammation as a conditioning approach is that MSCs will be primed to the environment into which they will be introduced. Here, we used rat MSCs with the aim of setting the ground for in vivo allotransplant preclinical studies. Our interpretations make reference to potential clinical applications because rat, mouse and human MSCs have been shown to behave similarly when exposed to stimuli activating the abovementioned pathways [27,28] and we consider it a translatable effect. A comparative analysis of the inflammatory conditioning strategy on rat, mouse and human cells would help understand whether the enhanced paracrine activity is conserved. We also showed that inflammatory conditioning may challenge MSC viability and motility. The impact of these possible effects of inflammatory conditioning on the pro-regenerative potential of MSCs in vivo is currently unknown. Additional preclinical research is needed to further explore the physiological effect of pre-conditioned MSCs and optimize the inflammatory conditioning method to most efficiently benefit from conditioning approaches that enhance the therapeutic potential of MSCs.

## 4. Materials and Methods

### 4.1. Animals

This study used adult female Sprague–Dawley rats (n = 6, 225–250 g, Charles Rivers Laboratory, Wilmington, MA, USA). We followed the guidelines of the National Institutes of Health and the United States Department of Agriculture for all animal procedures. The University of Miami Institutional Animal Care and Use Committee approved the procedures (Protocol #15-231, approved on 7 December 2015). The Assessment and Accreditation of Laboratory Animal Care accredited the animal facility. Rats were kept in pairs under a 12 h light/dark cycle with food and water accessible ad libitum.

### 4.2. Bone Marrow Harvest and Culture

Rats were euthanized in a CO_2_ chamber and their hind limbs immediately shaved and cleaned with antiseptic soap solution, followed by ethanol. The femurs and tibias were dissected out, rinsed and kept in ice-cold Leibovitz’s L-15 medium (ThermoFisher Scientific, Waltham, MA, USA). From the bones, the epiphyses were cut off and, using a syringe and needle, the medullary cavity of the diaphysis flushed out with D10 medium, which consists of Dulbecco’s Modified Eagle medium (DMEM; ThermoFisher Scientific, Waltham, MA, USA) supplemented with glutamine, 10% fetal bovine serum (FBS), and 1:1000 gentamycin. The flushed-out marrow was suspended in D10 using a glass pipette and washed twice in D10 by centrifugation at 1500 revolutions per minute (rpm) for 5 min at 4 °C. The supernatants were discarded after the washes and the final pellet was suspended in D10 and strained through a 100 μm FalconTM cell strainer (BD Life Sciences, East Rutherford, NJ, USA) to remove debris. The final cell suspension was added to a 100 mm uncoated plastic FalconTM culture dish (BD Life Sciences, East Rutherford, NJ, USA) and cultured at 37 °C and 6% CO_2_ for 16-24 h. In the culture dish, monocytes were suspended in the culture medium and MSCs adhered to the bottom. The culture supernatant was collected after 20 to 24 h and ran through fluorescence-activated cell sorting (FACS) to separate the monocytes. Fresh D10 was added to the MSCs that adhere to the plastic dish within the first 10 to 20 h of culture. With this protocol, we obtained MSC cultures and macrophage cultures from the same source bones.

### 4.3. Mesenchymal Stromal Cells

The bone marrow-derived MSCs were cultured on plastic for 72 h. After discarding the medium, attached cells were washed twice with Hank’s Balanced Salt Solution (HBSS; ThermoFisher Scientific, Waltham, MA, USA) and then detached with Trypsin-EDTA in HBSS for 3 min. A glass pipette was used to suspend the MSCs, which were then collected in a 50 mL conical tube with D10 and centrifuged at 1500 rpm for 5 min at 4 °C. Each conical tube contained MSC suspensions from one rat. The pellet was resuspended and washed once in D10 to remove and neutralize the remaining trypsin. The cells were then plated on poly-D-lysine (PDL)-coated dishes with D10 and labeled passage 0 (P0). Medium was refreshed every 72 h and near confluent cultures were split into the next passage. At P2, FACS was used to sort out MSCs and remove mature macrophages, which are present in bone marrow and attach to plastic during the first 8 h of culture. Sorted MSCs were cultured in D10 on PDL-coated dishes until P4. The mesenchymal phenotype of P4 MSCs was validated using flow cytometry (Supplementary Data Section I; Figure S2A–D). We used P4 MSCs in the experiments.

### 4.4. Macrophages

Bone marrow-derived monocytes were cultured, after sorting, in R10-50 medium (Roswell Park Memorial Institute-1640 medium (RPMI-1640; ThermoFisher Scientific, Waltham, MA, USA) with glutamine, 10% fetal bovine serum, 1:1000 gentamycin and 50 ng/mL macrophage colony stimulating factor (M-CSF; Peprotech Inc., Rocky Hill, NJ, USA) in uncoated plastic FalconTM culture dishes (BD Life Sciences, East Rutherford, NJ, USA) for 7 days to induce differentiation into macrophages. On day 3, R10-50 was refreshed. On day 7, some of the macrophage cultures received fresh R10 to maintain the macrophages as non-polarized macrophages (M0). The remaining cultures received R10-50 supplemented with 100 ng/mL of bacterial lipopolysaccharide (LPS; Sigma-Aldrich, St Louis, MO, USA) and 25 ng/mL of rat recombinant interferon-gamma (IFNγ; Peprotech Inc., Rocky Hill, NJ, USA) and were incubated for 24 h to polarize the macrophages to a classic pro-inflammatory phenotype (M1). The culture media of the M0 and M1 polarized macrophages were collected and centrifuged at 1500 rpm for 5 min at 4 °C to remove cellular debris. These provided the M0 (M0CM) and M1 conditioned media (M1CM) used in our experiments to condition MSCs. The polarized phenotype of M0 and M1 macrophages was confirmed using immunocytochemistry and fluorescence imaging (Supplementary Data Section I; Figure S2E–J).

### 4.5. Flow Cytometry Cell Sorting

MSCs obtained from confluent P2 cultures were trypsinized and quantified using the CountessTM II FL automated cell counter (ThermoFisher Scientific, Waltham, MA, USA). MSCs were stained with CD45-AF647 (#202212, BioLegend, San Diego, CA, USA), CD29-PE (#12-0291-81, ThermoFisher Scientific, Waltham, MA, USA) and the dead cell stain SYTOX Green (#S34862, ThermoFisher Scientific, Waltham, MA, USA), and then sorted using a Beckman Coulter MoFlo Astrios EQ cell sorter (Beckman Coulter Inc., Pasadena, CA, USA) operated with a 100 um nozzle, at 25 psi, and an event rate of approximately 10,000 events per second. Sorted MSCs were further cultured until P4.

Monocytes, to be sorted from the suspension of non-adherent bone marrow cells, were washed by centrifugation and counted using the CountessTM II FL automated cell counter. The cell suspension was incubated with CD45-AF647 (#202212, BioLegend, San Diego, CA, USA) and CD11b/c-PE (#12-0110-82, eBioscience Inc., San Diego, CA, USA), and the dead cell stain SYTOX Green (#S34862, ThermoFisher Scientific, Waltham, MA, USA).

All cells (107 cells/mL) were stained with antibodies and dyes for 25 min on ice, washed twice, and resuspended in custom flow buffer containing HBSS (non-Phenol Red), 25 mM HEPES, 0.1% gentamycin, and 5% fetal bovine serum. Antibodies were used at the concentrations for flow cytometry provided by the manufacturers.

### 4.6. MSCs Conditioning

MSCs were conditioned when cultures were about 80% confluent at P4. Macrophage-derived conditioned media (M0CM and M1CM) were thawed in a 37 °C water bath and gently mixed. D10 medium (D10) and macrophage polarization medium (Pol1) were freshly prepared and warmed up to 37 °C. MSC cultures were rinsed with D10 before conditioning with M0CM, M1CM, Pol1, or D10, for 24 h at 37 °C and 6% CO_2_. After conditioning, culture media were collected, centrifuged at 1500 rpm for 15 min to remove cellular debris, aliquoted, and stored at −80 °C.

### 4.7. Immunoassays

The level of nerve growth factor (NGF), human growth factor (HGF), glial-derived neurotrophic factor (GDNF), and vascular endothelial growth factor (VEGF) in the medium of conditioned MSCs and in the conditioning media (M0CM, M1CM, Pol1, D10) were determined using absorbance-based sandwich ELISAs, following the manufacturers’ protocols. Samples were thawed at room temperature, vortexed, spun down at 1000 G for 20 min at 4 °C, and assayed using NGF beta rat ELISA Kit (#ERNGF, ThermoFisher Scientific, Waltham, MA, USA), Rat HGF ELISA kit (#MBS825055, MyBiosource Inc., San Diego, CA, USA), Rat GDNF PicoKineTM ELISA Kit (#EK0363, Boster Bio, Pleasanton, CA, USA), and Rat VEGF Quantikine ELISA Kit (#RRV00, R&D Systems, Bio-techne, Minneapolis, MN, USA). Absorbance readings were carried out in a FLUOStar Omega microplate reader (BMG Labtech, Ortenberg, Germany).

### 4.8. RNA Extraction, Library Preparation, and Sequencing

RNA was extracted from MSCs after trypsinization following the manufacturer’s protocol for the RNeasy Mini Kit (Qiagen, Hilden, Germany) (further protocol details in Supplementary Data Section II). The samples, diluted to the desired concentration with RNAse-free water, were shipped to Novogene USA (Novogene Co., Beijing, China) for RNA sequencing and data analysis. Novogene controlled the sample quality using NanoDropTM, Agilent 2100 Bioanalyzer (Agilent Technologies Inc., Santa Clara, CA, USA) and agarose gel electrophoresis to measure the RNA integrity number (RIN) and sample purity.

cDNA libraries were built from a small amount of RNA using Illumina kits (Illumina Inc., San Diego, CA, USA) and quality controlled (QC) for concentration (Qubit 2.0 fluorometer; Life Technologies, Carlsbad, CA, USA) and insert size (Bioanalyzer). RNA quantity was confirmed at Novogene USA by quantitative polymerase chain reaction (qPCR). Sequencing was performed with the Illumina Platform PE150 (Q30 ≥ 80%), obtaining an average of 30 M reads/sample. The resulting FASTQ files were quality controlled for the rate of base calling error, A/T/G/C content distribution, and cleanliness.

### 4.9. Sequence Annotation and Differential Gene Expression

Clean reads were mapped to the rat reference genome (Rnor 6.0) using the TopHat algorithm (v2.0.12; mismatch = 2). We determined the regions mapped and the total, multiple, and distribution of mapped reads per chromosome to assess the quality of the data set. After confirmation of the good quality of the mapped reads, gene expression was quantified from the transcripts that mapped to exons using the HTSeq algorithm (v0.6.1; -m union). Gene expression levels were expressed in fragment per kilobase of transcript sequence per millions base pair sequenced (FPKM), which accounts for sequencing depth and gene length [97]. Plotting the FPKM mean of the replicates in a violin plot enabled the comparison and validation of the distribution of gene expression across conditions. Correlations were used to validate the reliability of the biological replicas within and across conditions. We applied the normalized quantification data to a statistical model (DESeq package, v1.10.1) to calculate differential expression (DE) of genes and the *p*-value. The value of false discovery rate (FDR), corrected for multiple testing to adjust the *p* value (padj). Genes with a padj < 0.05 were considered differentially expressed genes (DEG) and used for downstream analysis. Quantification, DE analysis, and data representation were conducted on language and statistical environment R (RStudio Team, Boston, MA, USA). A cluster analysis was used to visualize the relationships and patterns of gene expression across conditions, based on the log10(FPKM + 1) value. The raw and processed data generated during this RNAseq study were uploaded to the GEO repository under accession number GSE161798 (https://www.ncbi.nlm.nih.gov/geo/query/acc.cgi?&acc = GSE161798, accessed date: 20 November 2020).

### 4.10. Gene Ontology and KEGG Pathway Analyses

The biological meaning of the DEG was determined using the gene ontology (GO) and KEGG databases. A hypergeometric test run with the GOseq tool (v2.12) was used to determine GO term enrichment. The enrichment of a GO term is the ratio between the number of DEG annotated for a specific GO term in our dataset and the number of reference genes for that GO term in the database. The hypergeometric test also determined the probability that the association to a GO term was made by chance [98]. GO terms are used to describe biological process, cellular components, and molecular function annotations.

The KOBAS tool (v3.0) was used to determine biological pathways annotated to the DEG in our dataset and calculated the level of enrichment and significance of each pathway by hypergeometric test. The level of enrichment of a biological pathway was represented here by the rich factor (RF), which is the ratio between the DEG annotated to a pathway and the number of reference genes for that KEGG pathway. The threshold of significance for both enrichments was set to padj < 0.05.

### 4.11. Statistical Analysis

Secretome data were statistically analyzed using SPSS Statistics package 26 (IBM, Armonk, NY, USA). Data were tested for normality before running statistical tests. One-way ANOVA was used to compare data between groups when the data were normally distributed. Kruskal–Wallis was used to compare data between groups when the data were not normality distributed. The threshold of significance was *p* < 0.05. Bonferroni correction was used for post hoc testing. GraphPad PRISM 8 (GraphPad Software Inc., San Diego, CA, USA) was used for data visualization.

## Data Availability

The data presented in this study are openly available in the NIH Gene Expression Omnibus (GEO) repository, under accession number GSE161798.

## Supplementary Materials

Supplementary materials can be found at https://www.mdpi.com/1422-0067/22/2/781/s1.

## Abbreviations

MSC	Mesenchymal stromal cell
RNAseq	RNA high throughput sequencing
GO	Gene ontology
LPS	Lipopolysaccharide
D10	Baseline medium for MSC (DMEM + fetal bovine serum + gentamycin)
Pol1	Pro-inflammatory polarization medium for macrophages
M0CM	Conditioned medium from non-polarized macrophages (M0)
M1CM	Conditioned medium from pro-inflammatory macrophages (M1)
IFNγ	Interferon gamma
FACS	Fluorescence activated cell sorting
P2, P4	Passage 2 or 4
CM	Conditioned medium
PDL	Poly-(D)-Lysine
FPKM	Fragments Per Kilobase of transcript sequence per Millions base pairs sequenced
DEG	Differentially expressed gene
DE	Differential expression
BP	Biological process
MAPK	Mitogen activated protein kinase
*hif1α*	Hypoxia inducible factor 1 subunit alpha
*nfkb1*	Nuclear factor kappa B subunit 1
*nfkbiz*	Nuclear factor kappa B inhibitor zeta
*hgf*, HGF	Hepatocyte growth factor
*ngf*, NGF	Nerve growth factor
*ido1*	Indoleamine 2,3-dioxygenase 1
*irfs*	Interferon regulatory factors
DAG	Directed acyclic graph
*p*(adj)	*p*-value adjusted for multiple testing
CC	Cellular component
MF	Molecular function
MHC	Major histocompatibility complex
KEGG	Kyoto Encyclopedia of Genes and Genomes
RF	Rich factor
TNF	Tumor necrosis factor
*Gdnf*, GDNF	Glial derived neurotrophic factor
*Vegf*, VEGF	Vascular endothelial growth factor
*bcl2*	B cell lymphoma 2
JAK	Janus kinase
STAT	Signal transducer and activator of transcription
ELISA	Enzyme-linked immunosorbent assay
*nos2*	Nitric oxide synthase 2
*il13r*	Interleukin 13 receptor
*ptgs2*	Prostaglandin synthase 2
*ang-1*	Angiopoietin 1
*egf*	Endothelial growth factor
IGF	Insulin like growth factor
FGF	Fibroblast growth factor
*bmp*	Bone morphogenetic protein
*wnt*	Wingless-Int-1 protein family
PGE2	Prostaglandin E2
QC	Quality control
GEO	Gene expression omnibus
