# Supplementary material for: Macrophage-Derived Inflammation Induces a Transcriptome Makeover in Mesenchymal Stromal Cells Enhancing Their Potential for Tissue Repair
Source: Int J Mol Sci. 2021 Jan 14;22(2):781. doi: 10.3390/ijms22020781 (PMC7828776; doi:10.3390/ijms22020781)
Supplement: Supplementary file 1 [file ijms-22-00781-s001.zip › RevisedIJMS_RNAseq Paper Supplementary data.docx]

**Supplementary material**

**Macrophage-derived inflammation induces a transcriptome makeover in mesenchymal stromal cells in support of tissue repair**

Maldonado-Lasunción I*, O’Neill N, Umland O, Verhaagen J, Oudega M*

**Table of contents**

**Section I – Cell characterization.** Includes the methods to evaluate MSC mesenchymal phenotype and macrophage polarization, their results and **Fig S1**.

**Section II – RNA extraction and quality control.** Includes extended methods for RNA extraction and results of RNA extraction and quality evaluation.

**Section III – Sequencing overview and quality control.** Includes **Fig S2,** **Table S1** and **Table S2.**

**Section IV – Gene Ontology Analysis**. Includes **Table S3**, **Table S4** and **Table S5**.

**Section V – KEGG Pathway Analysis**. Includes **Table S6**, **Table S7** and **Fig S3**.

*Table S1 and Tables S3 - S7 are attached as separate spreadsheets.*

*Figures S1-S3 are included in full resolution in the zip folder with the main manuscript figures.*

**Section I – Cell characterization.**

**Methods for flow cytometry for MSC**

P4 MSC were characterized using a CytoFLEX flow cytometer (Beckman Coulter Inc, California, USA). For this, trypsinized MSC were counted using the Countess^TM^ II FL automated cell counterand resuspended to a single cell suspension. The cell suspension was incubated with CD29-PE (#12-0291-81, ThermoFisher Scientiifc), CD90-FITC (#ab226, Abcam, Cambridge, United Kingdom), CD34-AF647 (#NBP2-33076-AF647, Novus Biologicals, Colorado, USA), CD45-PE-Vio770 (#130-107-791, Miltenyi Biotec, Bergisch Gladbach, Germany) and the cell viability dye Ghost Dye Red 780 (#13-0865-T100, Tonbo Biosciences, California, USA), then screened on the flow cytometer. Cells (10^7^ cells/ml) were incubated with the antibodies and viability dye for 25 min on ice, washed twice, and then resuspended in custom flow buffer containing HBSS (non-Phenol Red), 25 mM HEPES, 0.1 % gentamycin, and 5 % fetal bovine serum. Antibodies were used at the concentrations for flow cytometry provided by the manufacturers.

**Methods for immunocytochemistry and imaging**

We characterized the polarized macrophages using immunocytochemistry. After polarization and CM collection, macrophages were washed and fixed with 4 % paraformaldehyde for 10 min at room temperature. The cells were then washed and incubated in PBS with 5 % normal goat serum (Vector Laboratories Inc, California, USA) and 0.3 % Triton X-100 for 1 h at room temperature to block non-specific binding and permeabilize the cells. For staining, we used primary antibodies against a general macrophage marker, CD68 (1:200; ED1 clone, #MAB1435, MilliporeSigma, Massachusetts, USA) and against the classic inflammatory marker, inducible nitric oxide synthase (prediluted; iNOS; #ab15326, Abcam, Cambridge, United Kingdom) in PBS with 5 % normal goat serum (Vector Laboratories Inc, California, USA) and 0.3 % Triton X-100 for 2 h at room temperature. The cells were washed three times in PBS and then incubated with the secondary antibody goat-anti-mouse IgG-AF555 (1:500; #ab150114, Abcam) and goat-anti-rabbit IgG-AF488 (1:500; #A-11034, ThermoFisher Scientific) in PBS for 2 h in the dark at room temperature. After two washes with PBS, the cells were imaged with a Zeiss AxioVert 200M inverted fluorescence microscope (Carl Zeiss AG, Oberkochen, Germany).

**Results of cell characterization**

The mesenchymal phenotype of sorted MSC was studied by flow cytometry using fluorescently labeled antibodiesto cell surface antigens (Fig. S1). MSC were positive for CD90 (99.6%) and CD29 (99.9%), and negative for CD45 (0.7%) and CD34 (0.1%) (Fig. S1A). The average viability of trypsinized MSC used for RNA extraction was 96% across conditions (data not shown). The phenotype of the polarized pro-inflammatory macrophages, which we refer to as M1 macrophages, and the non-activated macrophages, which we refer to as M0 macrophages, was studied using staining for iNOS/CD68 and CD68 only, respectively (Fig. S1B). Thus, our data showed that the MSC cultures used for the experiments were of high purity and that the macrophages were properly polarized.


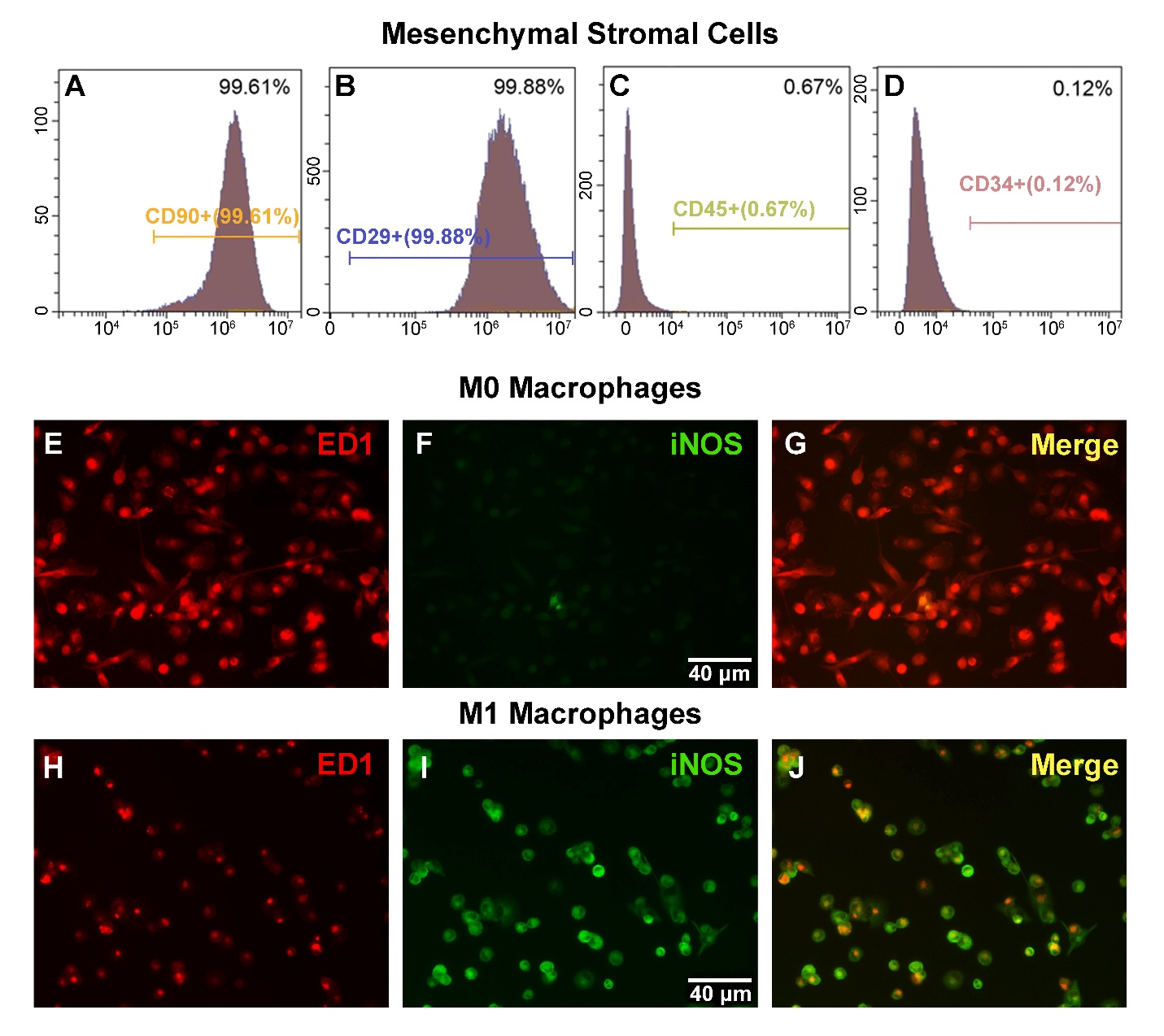


**Fig. S1. Cell characterization.** Mesenchymal stromal cells purity was confirmed by flow cytometry based on the expression of CD90, CD29 and the absence of CD45 and CD34. **(A)** 99.61% of cells were positive for marker CD90; **(B)** 99.88% of cells were positive for marker CD29; **(C)** 0.67% of cells were positive for marker CD45, which is considered as a culture negative for CD45; **(D)** 0.12% of cells were positive for marker CD34, which is considered as a culture negative for CD34. Macrophages were properly polarized to pro-inflammatory (M1) or left unpolarized (M0), as shown here by ICC. Photomicrographs of fixed cells showing fluorescently immunostained M0 macrophages **(E)** positive for CD68 (ED1), a general macrophage surface marker, **(F)** negative for iNOS, a canonical pro-inflammatory marker **(G)** and the merge of the two stainings; and M1 macrophages **(H)** positive for CD68 (ED1), **(I)** positive for iNOS **(J)** and the merge of the two stainings. Scale bar is 40 μm.

**Section II – RNA extraction, quality control and qPCR.**

**Methods for RNA extraction and quality evaluation**

After cell lysis with RLT and addition of the samples to the spin columns, a DNase step was performed using a DNase set (Qiagen, Hilden, Germany) to eliminate DNA contamination. RNA was purified on the spin column and eluted with RNase-free water. RNA concentration and purity were tested with a NanoVue^TM^ Plus Spectrophotometer (GE Healthcare, Illinois, USA). Finally, samples were diluted to the desired concentration with RNase-free water.

**Results of RNA extraction and RNA quality**

After 24 h conditioning, we extracted 32.4 ± 7.5 μg RNA from the cultures. The extracted RNA had a A260/A280 ratio of 2.1 ± 0.006 and a A260/A230 ratio of 2.3 ± 0.11. The RIN of the extracted RNA was 10 across all samples, according to the Bioanalyzer and agarose gel electrophoresis data. Note that RNA quality control was performed at each step during the experiments, sequencing and just prior to DE analysis. Together, the data showed that the extracted RNA was of high quality and purity, therefore suitable for preparing libraries and performing sequencing.

**Section III – Sequencing overview and quality control**


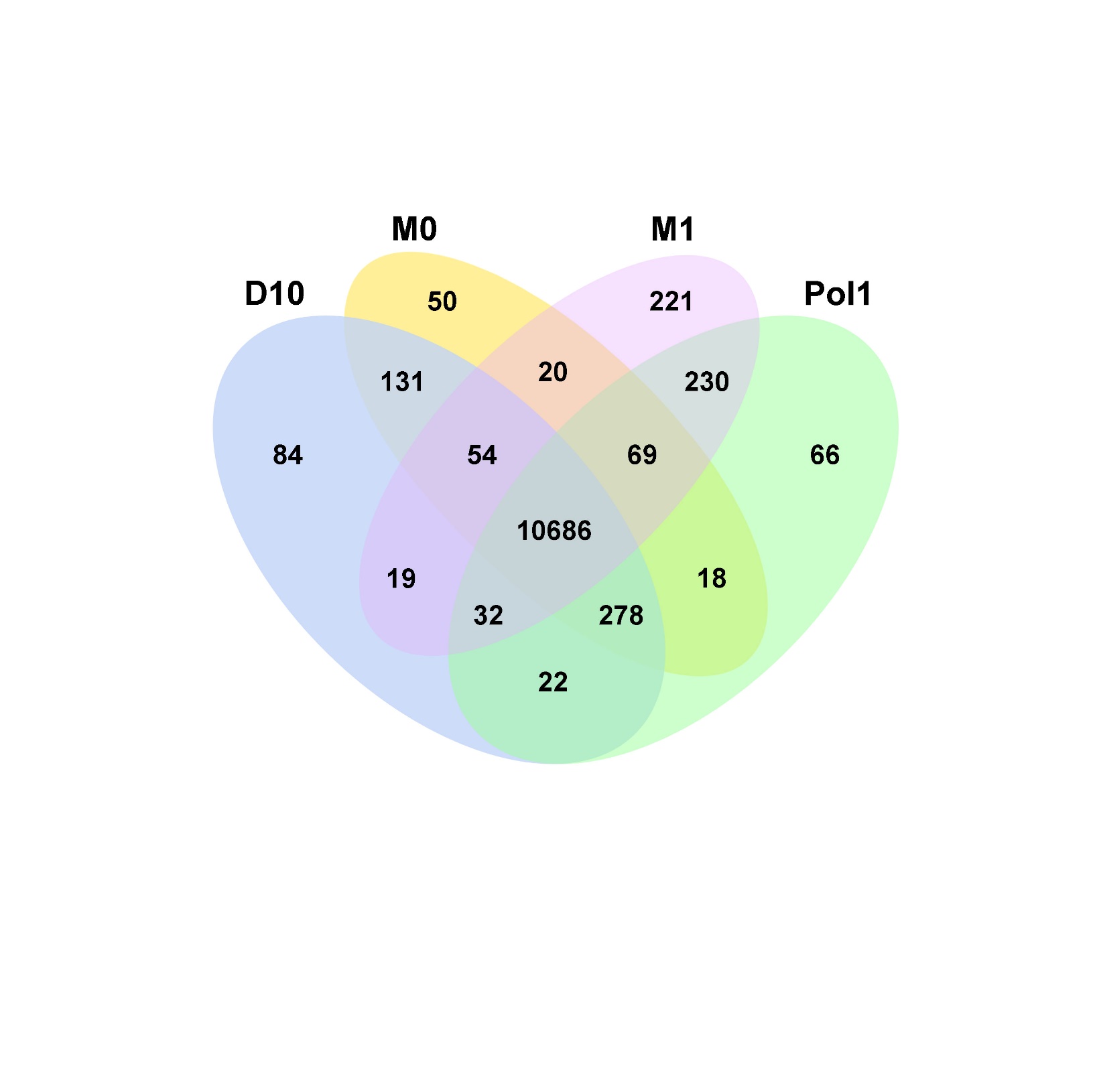


**Fig. S2. Co-expression Venn diagram.** Venn diagram showing the number of genes expressed across conditions and the overlap between them. The numbers present in sections covering just one condition represent the number of genes expressed uniquely in that condition, while the areas representing overlap between ellipses contain the numbers for the genes expressed in two or more of those conditions. The sum of the numbers within one ellipse represent the number of total genes expressed in that condition.

**Table S1:** **Mapping quality summary.**

*Detailed sample information included in separate spreadsheet with full Table S1.*

| **Sample name** | **Average** | **Std Deviation** |
| --- | --- | --- |
| **Total reads ^a^** | 72098228.63 | 6472765.53 |
| **Total mapped (%) ^b^** | 86.66 | 1.27 |
| **Multiple mapped (%) ^c^** | 4.70 | 0.57 |
| **Uniquely mapped (%) ^d^** | 81.96 | 1.52 |
| **Read-1 (%)** | 41.33 | 0.80 |
| **Read-2 (%)** | 40.63 | 0.75 |
| **Reads map to '+' (%) ^e^** | 40.93 | 0.76 |
| **Reads map to '-' (%) ^e^** | 41.04 | 0.77 |
| **Non-splice reads (%) ^g^** | 37.70 | 1.18 |
| **Splice reads (%) ^g^** | 44.26 | 1.56 |

^a^ Total number of filtered reads (Clean data).

^b^ Total number of reads that can be mapped to the reference genome. In general, this number should be larger than 70% when there is no contamination and the correct reference genome is chosen.

^c^ Number of reads that can be mapped to multiple sites in the reference genome. This number is usually less than 10% of the total.

^d^ Number of reads that can be uniquely mapped to the reference genome.

^e^ Number of reads that map to the positive strand (+) or the minus strand (-).

^f^ Splice reads can be segmented and mapped to two exons (also named junction reads), whereas non-splice reads can be mapped entirely to a single exon. The ratio of splice reads depends on the insert size used in the RNA-seq experiments.

**Table S2:** **Summary of differentially expressed genes between conditions.**

| **Comparison** | **Upregulated DEG^a^** | **Downregulated DEG^b^** |
| --- | --- | --- |
| **M1 vs D10** | 1721 | 1487 |
| **M0 vs D10** | 54 | 5 |
| **Pol1 vs D10** | 919 | 509 |
| **M1 vs Pol1** | 813 | 588 |
| **M1 vs M0** | 1535 | 1312 |
| **M0 vs Pol1** | 452 | 776 |

The total number of up-^a^ or down-regulated^b^ DEG resulting from the comparison between conditions. The criteria to identify DEGs was a fold change > 0 and the statistical significance of the change in gene expression p(adj) < 0.05. Abbreviations: DEG: differentially expressed genes; D10: MSC conditioned with DMEM medium with 10% fetal bovine serum; M0: MSC conditioned with M0CM; M1: MSC conditioned with M1CM; Pol1: MSC conditioned with pro-inflammatory polarization medium.

* The full list of DEG obtained per comparison is available in the GEO data repository, under accession GSE161798.

**Section IV – Gene Ontology Analysis.**

The GO analysis is presented in the main manuscript, but we considered it relevant to share the full list of significantly enriched GO terms for each term type. The GO terms are sorted in order of significance and the rich factor data is color coded for easier reference to the directionality of the enrichment (whether it is more enriched with up- or down-regulated genes).

**Table S3: Top 100 biological process GO terms resulting from M1 Vs D10 comparison.**

*Full table included in separate spreadsheet for Table S3.*

**Table S4: Cellular component GO terms resulting from M1 Vs D10 comparison.**

*Full table included in separate spreadsheet for Table S4.*

**Table S5: Molecular function GO terms resulting from M1 Vs D10 comparison**

*Full table included in separate spreadsheet for Table S5.*

**Column legend for tables S3 thru S5:**

(1) GO accession: Gene Ontology entry.

(2) Description: Detailed description of Gene Ontology.

(3) Over represented p-Value: p-value in hypergenometric test.

(4) Corrected p-Value: Corrected P-value; GO with corrected p-values < 0.05 are significantly enriched in DEGs.

(5) DEG item: Number of DEGs with GO annotation.

(6) Bg_item: Number of background genes related to this GO.

(7) Up: Number of up-regulated DEGs related to this GO.

(8) Down: Number of down-regulated DEGs related to this GO.

(9) Up_RF: Ratio of upregulated DEG over background genes related to that GO, representing the level of enrichment of this GO term with up_DEGs.

(10) Down_RF: Ratio of downregulated DEG over background genes related to that GO, representing the level of enrichment of this GO term with down_DEGs.

(11) Up_Gene_names: ID of up-regulated DEGs related to this GO.

(12) Down_Gene_names: ID of down-regulated DEGs related to this GO.

**Section V – KEGG Pathway Analysis.**

The KEGG analysis is presented in the main manuscript, but here we share the list of top 60 significantly enriched GO terms for each term type. The GO terms are sorted based on the rich factor data.

**Table S6: Biological pathways enriched with upregulated DEG**

*Full table included in separate spreadsheet for Table S6.*

**Table S7: Biological pathways enriched with downregulated DEG**

*Full table included in separate spreadsheet for Table S7.*

**Column legend for tables S6 and S7:**

(1) Database: The database of pathway.

(2) ID: KEGG ID.

(3) #Term: The description of KEGG pathways.

(4) Input number: Number of DEGs with pathway annotation.

(5) Background number: Number of all reference genes with pathway annotation.

(6) P-value: P-value in hypergenometric test.

(7) Corrected P-value: Corrected P-value，pathway with Corrected P-value < 0.05 are significantly enriched in DEGs.

(8) Rich factor: Ratio between upregulated DEG in our data and the number of genes assigned to that pathway in KEGG

(9) Input: Genes that related to the Pathway.

(10) KEGG_ID/KO: The KEGG_ID or KO number of DEGs enriched.

(11) Entrez_ID: The NCBI-GeneID of DEGs enriched.

(12) Hyperlink: the URL to the biological pathway diagram.

**Example of content included in tables S6 and S7:**


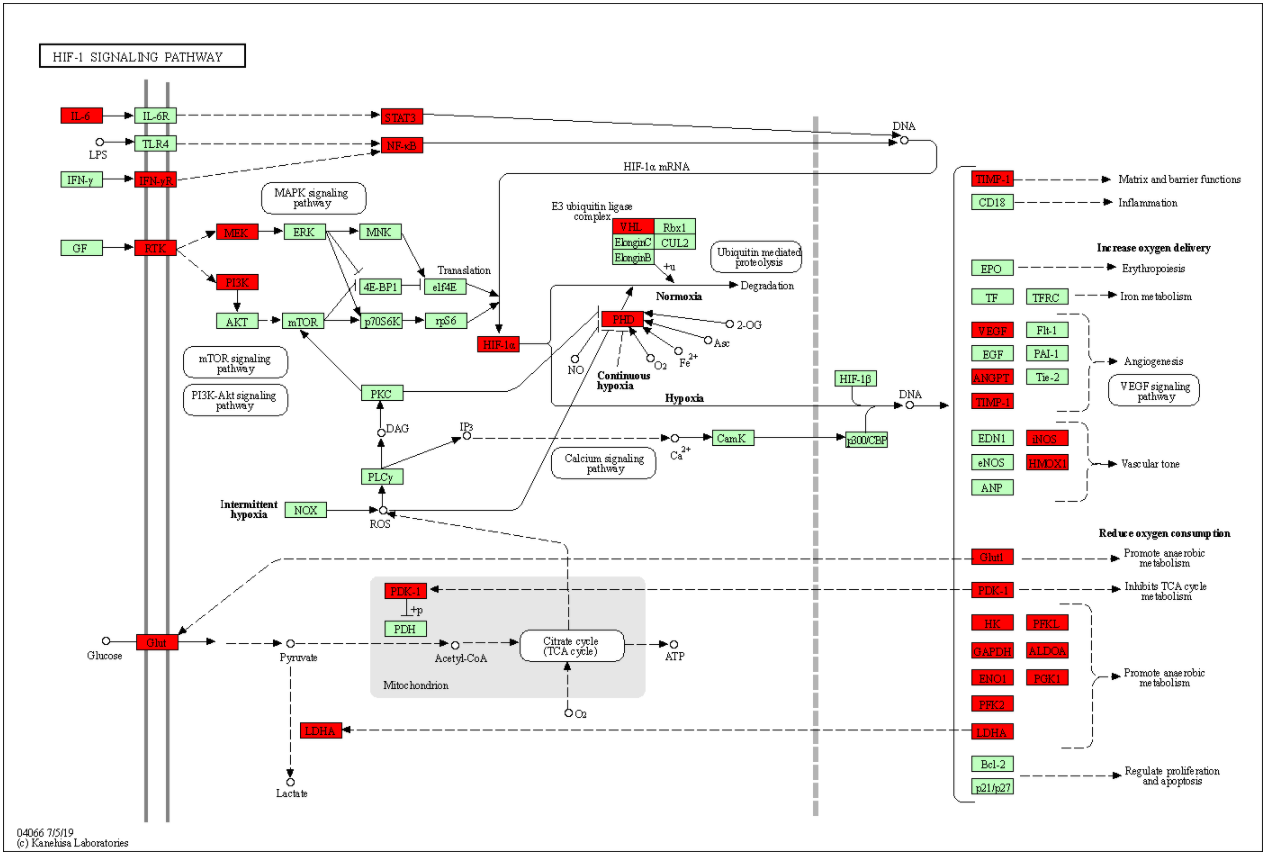


**Fig. S3. Snapshot of HIF-1 signaling pathway, an example resulting from following the URL hyperlink included on Tables S6 and S7.** The KEGG pathway analysis results include a hyperlink with each pathway entry, where we can observe the genes annotated to that pathway in the database, details about gene variants and color coded to represent gene expression in our experiments. The red boxes contain upregulated DEG in our dataset and the green boxes downregulated genes. If a box contains up- and downregulated genes, it will be represented in yellow.
