# Supplementary figures and images for: Macrophage-Derived Inflammation Induces a Transcriptome Makeover in Mesenchymal Stromal Cells Enhancing Their Potential for Tissue Repair
Source: Int J Mol Sci. 2021 Jan 14;22(2):781. doi: 10.3390/ijms22020781 (PMC7828776; doi:10.3390/ijms22020781)

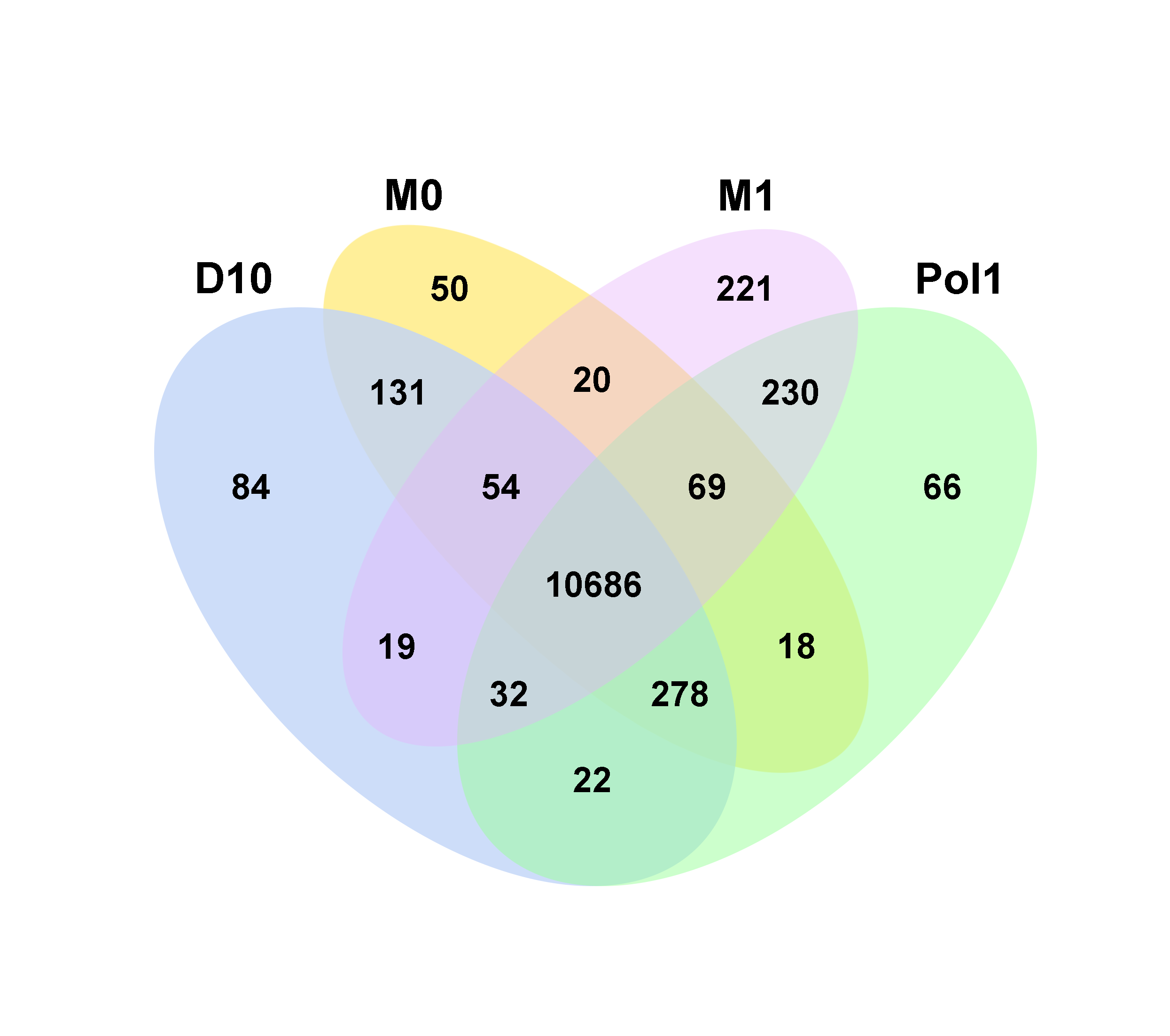

Supplement: Supplementary file 1 [file ijms-22-00781-s001.zip › RNAseqSubmission-Figure S1.tif]

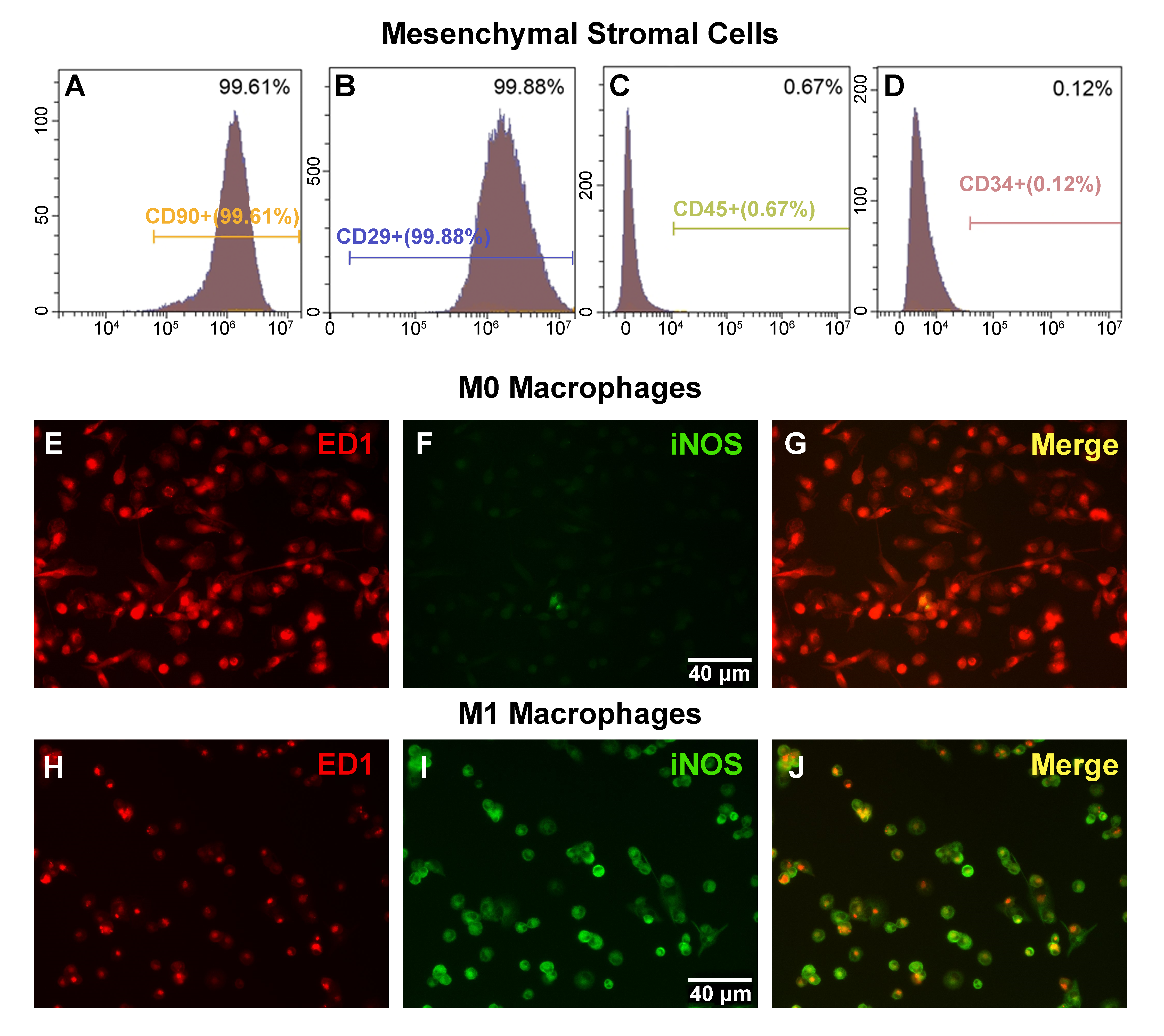

Supplement: Supplementary file 1 [file ijms-22-00781-s001.zip › RNAseqSubmission-Figure S2.tif]

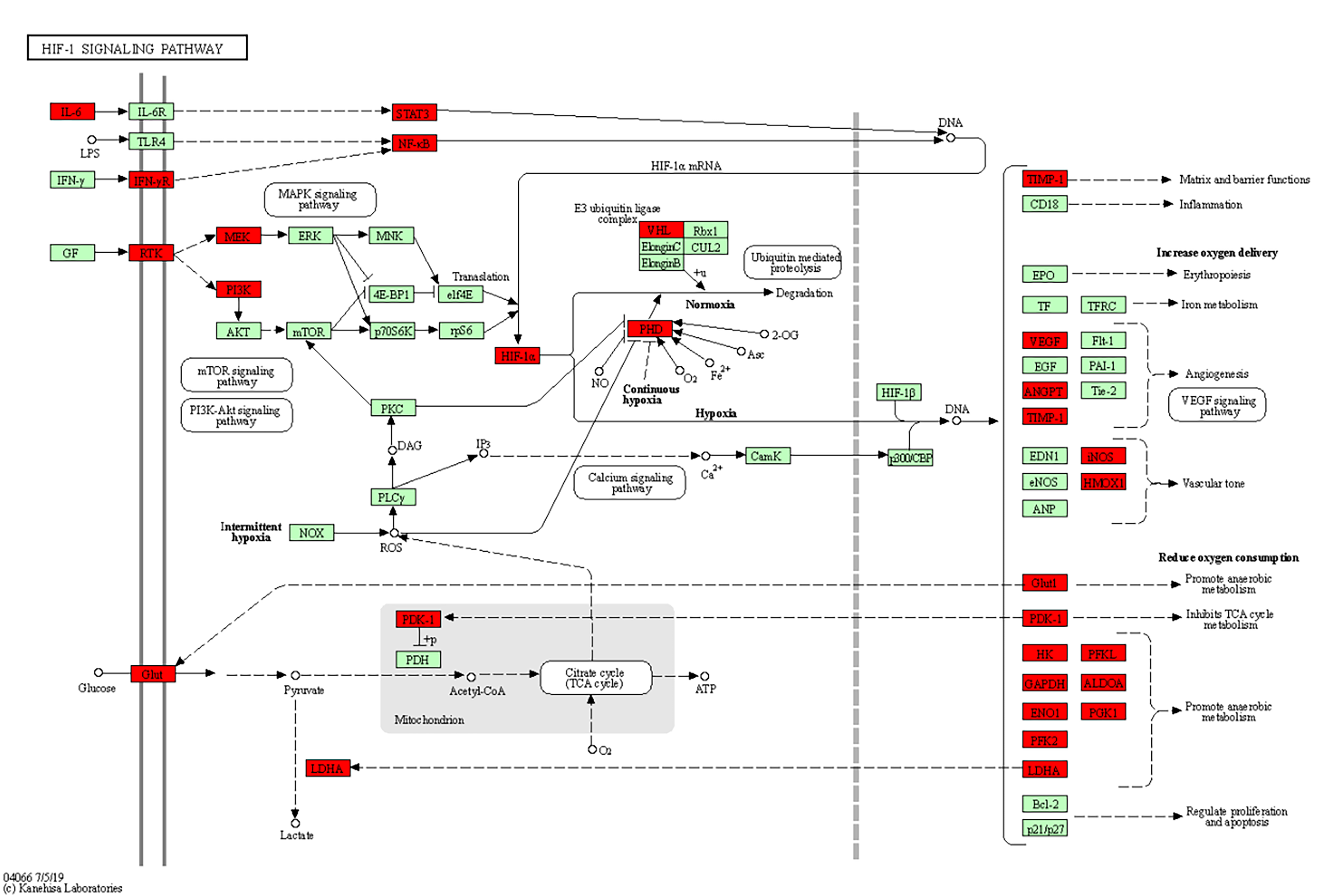

Supplement: Supplementary file 1 [file ijms-22-00781-s001.zip › RNAseqSubmission-Figure S3.tif]
